# Increasing proline and myo-inositol improves tolerance of *Saccharomyces cerevisiae* to the mixture of multiple lignocellulose-derived inhibitors

**DOI:** 10.1186/s13068-015-0329-5

**Published:** 2015-09-15

**Authors:** Xin Wang, Xue Bai, Dong-Fang Chen, Fu-Zan Chen, Bing-Zhi Li, Ying-Jin Yuan

**Affiliations:** Key Laboratory of Systems Bioengineering (Ministry of Education), Tianjin University, Tianjin, 300072 People’s Republic of China; SynBio Research Platform, Collaborative Innovation Center of Chemical Science and Engineering (Tianjin), School of Chemical Engineering and Technology, Tianjin University, Tianjin, 300072 People’s Republic of China; School of Management and Economics, Tianjin University, Tianjin, 300072 People’s Republic of China

**Keywords:** Synthetic biology, Metabolomics, Tolerance, Lignocellulose-derived inhibitors, Proline, Myo-inositol

## Abstract

**Background:**

The development of robust microbes with tolerance to the combined lignocellulose-derived inhibitors is critical for the efficient cellulosic ethanol production. However, the lack of understanding on the inhibition mechanism limited the rational engineering of tolerant strain. Here, through the metabolomic analysis of an adaptation process of *Saccharomyces cerevisiae* to representative inhibitors, i.e., furfural, acetic acid and phenol (FAP), we figured out the new candidates for improving inhibitor tolerance.

**Results:**

After metabolomic analysis, proline and myo-inositol were identified as the potential metabolites responsible for strain tolerance to inhibitors. The deletion of genes involved in proline or myo-inositol synthesis weakened strain tolerance against FAP stress. On the contrary, the addition of proline or myo-inositol in medium exerted a protective effect on cell growth under FAP stress. Furthermore, the enhancement of proline or myo-inositol synthesis by overexpressing key gene *PRO1* or *INO1* conferred yeast strain significantly increased FAP tolerance. All the recombinant strains finished the fermentation within 60 h under FAP stress, while the control strain was still in the lag phase. Meanwhile, it was found that the intracellular level of reactive oxygen species (ROS) under FAP condition was decreased with the increase of proline content, suggesting the function of proline as a ROS scavenger to protect strains from inhibitor damage.

**Conclusion:**

Increasing proline and myo-inositol were uncovered as the new determinants for improving strain tolerance to FAP under the guidance of metabolomics. Meanwhile, this study displayed the powerful application of metabolomics to develop rational strategies to increase stress tolerance and provided valuable insights into the design of recombinant microbes for the complex traits.

**Electronic supplementary material:**

The online version of this article (doi:10.1186/s13068-015-0329-5) contains supplementary material, which is available to authorized users.

## Background

With the rising concerns over the global petroleum supply and climate change, bio-based production of sustainable fuels from lignocelluloses has gained increased interest in the recent years [1]. However, large-scale production of biomass-derived fuels is still challenging. One of the major challenges is the numerous toxic compounds generated during the thermo-chemical pre-treatment process, such as furans, weak acids and phenolic compounds [2]. These inhibitory compounds severely inhibit cell growth and ethanol productivity, and ultimately diminish cost competitiveness [3, 4]. Therefore, it is urgent to develop the fermentation microbes with superior tolerance to such inhibitors for economically viable production of cellulosic ethanol.

Among these inhibitors, furfural, acetic acid and phenol are the three representations. In the previous study, researchers have made their efforts on the individual toxic mechanism of these inhibitors, and some yeast strains tolerant to single inhibitor have been developed [3, 4]. *S. cerevisiae* can convert furfural to the less toxic compound coupling with cofactors NAD(P)H catalyzed by multiple aldehyde reductases [5]. By overexpressing *ADH7* or *ADH1*, the yeast strains were successfully engineered with increased furfural resistance [6, 7]. At low pH, the undissociated acetic acid molecule could enter into cells across the plasma membrane and dissociates into acetate and protons in the cytoplasm, thus inducing intracellular acidification and inhibiting important metabolic processes [8]. To improve strain tolerance to acetic acid, the yeast strains were genetically modified through disrupting an aquaglyceroporin of the plasma membrane encoded by gene *FPS1* to decrease the uptake of extracellular acetic acid or overexpressing gene *HAA1* to reduce the intracellular accumulation of acetic acid [9, 10]. The yeast strain with enhanced tolerance to phenolic inhibitors was also developed by heterologous expression of laccase [11]. However, rare targets were reported for rationally improving strain tolerance to the mixture of these three representative inhibitors.

**Fig. 1:**
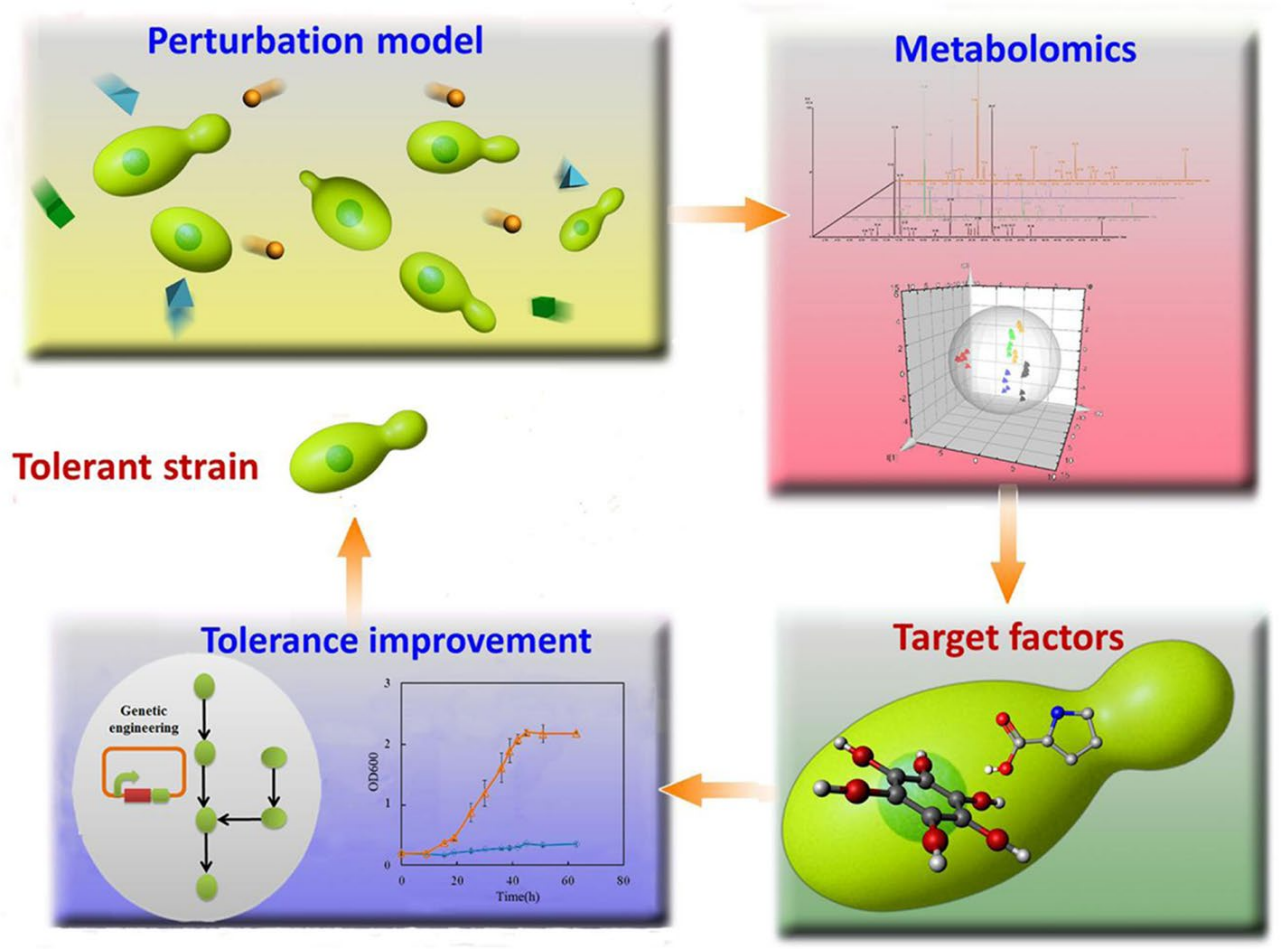
Schematic to improve microbial tolerance. The organism is perturbed by the toxic molecules and then metabolomic analysis is performed to figure out the potential biomarkers for the rational manipulation. The relevant genes were further modified to improve the strain tolerance

The metabolomic technique is a powerful tool to gain insight into the dynamic metabolic response to the exogenous or endogenous disturbance, which makes it possible to investigate genotype–phenotype relation [12]. It has been successfully used to identify the potential metabolites important for elucidating the molecular mechanism of specific biological systems [13, 14]. In this study, to improve strain tolerance to combined inhibitors (FAP; furfural, acetic acid and phenol), a comparative metabolomic analysis was performed on an adaptation process to figure out the potential biomarkers responsible for strain FAP tolerance (Fig. 1). Furthermore, the improved tolerance to multiple inhibitors was conferred by genetic modification of the relevant genes in *S. cerevisiae* (Fig. 1).

After the metabolomic analysis, the FAP sensitivity of the single-gene knockout mutants associated with the potential biomarkers was examined. It was observed that the deletion of genes involved in proline or myo-inositol synthesis increased strain sensitivity to multiple inhibitors, suggesting that proline or myo-inositol contents might be important for strain tolerance to FAP. Proline has been documented to be associated with resisting diverse stresses in a broad range of organisms, which could act as an osmolyte for osmotic adjustment, an oxidative stress protectant, a chemical chaperone, and a source of nitrogen and energy under nutrient limiting conditions [15, 16]. In *S. cerevisiae*, the intracellular myo-inositol content was found to affect strain tolerance against ethanol stress [17]. Based on our hypothesis, we found that exogenously added proline or myo-inositol improved cell growth under FAP stress. Meanwhile, the overexpression of *PRO1* or *INO1* gene in proline or myo-inositol biosynthetic pathway successfully increased strain tolerance to multiple inhibitors.

## Results and discussion

**Fig. 2:**
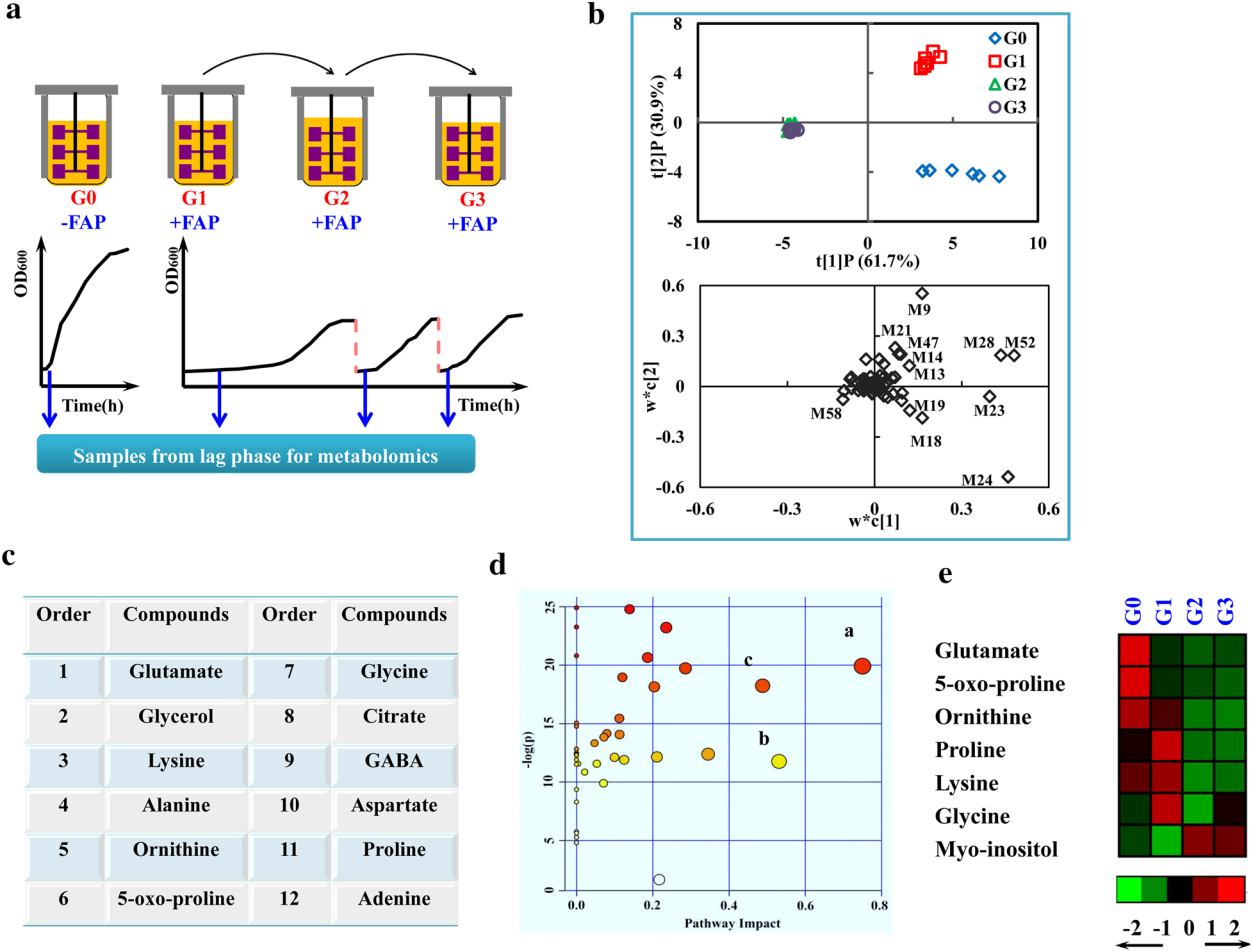
The discovery of potential biomarkers involved in strain tolerance to FAP through multivariate statistical analysis. **a** The schematic of the adaptation process and sampling strategy for metabolomic analysis. **b** PLS-DA score plot and loading plot of the samples from cells of *G0*, *G1*, *G2* and *G3*. The percentages listed in the axis labels described the fraction of variance explained by the first and second predictive component (t[1]P and t[2]P), respectively. *G0* cells without inhibitors, *G1* cells with inhibitors, *G2* and *G3* cells of transfer 1 and transfer 2. **c** The analysis results of minimum redundancy maximum relevance criterion (MRMR). **d** The metabolic pathway analysis with MetPA. All the matched pathways are displayed as *circles*. The *color and size* of each *circle* was based on *p* value (from pathway enrichment analysis), and pathway impact values (from pathway topology analysis), respectively. *a* Alanine, aspartate and glutamate metabolism; *b* glycerolipid metabolism; *c* arginine and proline metabolism. **e** Variations of the intermediates of proline synthesis metabolism, glycine, lysine and myo-inositol during the adaptation process. The levels of all metabolites were standardized by mean 0 and variance 1. The normalized level of each metabolite was indicated by the *color squares* at the *bottom right* of the map. The *bright red* and *green colors* represent the most elevated and reduced molecules

Complex phenotypes, such as strain tolerance to toxic compounds, are difficult to be rationally engineered due to the limited knowledge about molecular mechanism. Adaptation is a frequent method to gain insight into strain response to a specific stress condition [18]. In our work, to figure out the potential factors relevant to strain tolerance to combined inhibitors FAP, an adaptation experiment was carried out and thoroughly investigated by the metabolomic analysis. As shown in Fig. 2a, yeast cells were first cultivated in FAP-containing medium till stationary phase, and then an aliquot of the culture was transferred to fresh FAP-containing medium twice for additional rounds of growth. As the results described previously [19], in FAP-free medium, the cells in G0 entered the exponential phase after a transient lag phase. After the addition of FAP, the lag phase of cells in G1 was extended to 39 h, the fermentation time was delayed to 74 h from 40 h, and the final OD_600_ was reduced to 4.37 from 9.05 compared to cells of G0 in FAP-free medium. After transferring the cultures of G1 to next round, the cells of G2 rapidly adapted to combined inhibitors and started to grow only after 4 h in the lag phase [19]. The growth of cells in G3 was further slightly improved [19]. The glucose consumption was in consistence with cell growth and the final ethanol production was almost the same in all cultures. The duration of the lag phase could be interpreted as a measurement of varied tolerance to inhibitors [20, 21]. Meanwhile, the prolonged lag phase in adverse condition reflected a physiological shift of cells to adapt environmental stress. Therefore, samples for comparatively metabolomic study were collected at the lag phase of G0, G1, G2 and G3 (Fig. 2a). As shown in Additional file 1: Table S1, 70 putative intracellular metabolites were identified and quantified.

### The discovery of biomarkers associated with yeast tolerance to FAP by PLS-DA, mRMR and metabolic pathway analysis

For understanding and interpreting the adaptation model, the discovery of biomarkers is critical in the process of metabolomic analysis. Thus, appropriate statistical tools are essential for mining the huge data information. In this work, PLS-DA, mRMR and metabolic pathway analysis were carried out together to analyze our metabolite datasets. In the PLS-DA score plot (Fig. 2b), the first two predictive components explained 84.49 % of the total variance, while the first one accounted for almost 58.41 % alone. The clustering results revealed three major groups. The samples from G2 and G3 were clustered together, which were separated from G0 and G1 clearly, indicating the adjustment of intracellular metabolism of the yeast strain in response to FAP stimuli. In the PLS-DA loading plot (Fig. 2b), the intermediates of central carbon metabolism (glycerol and citrate), amino acids (glutamate, lysine, ornithine, 5-oxo-proline, alanine, aspartate, proline, glycine and GABA) and myo-inositol were identified as the most contributive metabolites in the separation of three groups, which were postulated to be important for strain FAP tolerance. In the results of mRMR analysis, the top 12 metabolites included the intermediates of central carbon metabolism (citrate and glycerol), amino acids (glutamate, lysine, ornithine, 5-oxo-proline, alanine, aspartate, proline, glycine and GABA) and adenine, which were all markedly affected in yeast cells during the adaptation to FAP (Fig. 2c). Among them, 11 of 12 biomarkers in mRMR were overlapped with those found in PLS-DA.

To further study the potential metabolic pathways involved in the yeast tolerance to FAP, the metabolic pathway analysis was performed using MetPA. The detected metabolites were correlated to 42 metabolic pathways, for instance, including pyrimidine metabolism, glutathione metabolism, and arginine and proline metabolism. It is well established that changes in the important positions of a network could trigger a more severe impact on the pathway than that in the marginal or relatively isolated positions [22]. Thus, only the out-degree for node importance measurements was considered here. The impact-value threshold was set to 0.40, above which the pathway was considered as the potential target pathway. Hence, we screened out three unique pathways potentially responsible for the improved inhibitor tolerance, including alanine, aspartate and glutamate metabolism, arginine and proline metabolism and glycerolipid metabolism (Fig. 2d). Meanwhile, the alanine, aspartate and glutamate metabolism and the arginine and proline metabolism were also significant in pathway enrichment analysis (Fig. 2d). Interestingly, the biomarkers alanine, aspartate, glutamate, GABA, and citrate located in the key positions in the alanine, aspartate and glutamate metabolic pathways, while the biomarkers proline, glutamate and ornithine were in the arginine and proline metabolic pathway. Taken together, we determined that alanine, aspartate and glutamate metabolism, arginine and proline metabolism, glycine, lysine, citrate, glycerol and myo-inositol might be strongly associated with yeast tolerance to FAP.

### The proposed targets relevant to FAP tolerance

The alanine, aspartate and glutamate metabolism and glycerol metabolism have been verified to be important for yeast cells to resist FAP stress in the previous study [23]. The metabolite citrate is one of TCA cycle intermediates. The significant variance of citrate level might be a suggestion that TCA cycle were markedly affected during the adaptation to FAP (Additional file 2: Figure S1). The metabolic flux analysis and comparative proteomics analysis showed that the addition of furfural could affect the activity of TCA cycle, which are involved in energy metabolism, as well as NADH production for the reduction of furfural [24, 25].

The detected intermediates (glutamate, proline, 5-oxo-proline and ornithine) in arginine and proline metabolic pathway were mainly involved in proline synthesis. Their significant variance reflected the marked effect of FAP on this pathway. As shown in Fig. 2e, the comparison of G0 and G1 showed that the intermediates of proline synthetic pathway including glutamate, 5-oxo-proline and ornithine were all significantly decreased when cells suffered sudden exposure to FAP, while the pathway end product of proline was increased by two times in cells of G1 as a response to FAP (Fig. 2e). It was consistent with the conclusion from the previous studies that proline was accumulated as a stress protectant in plants in response to various stress conditions [16]. With the adaptive evolution to FAP, the levels of proline and other intermediates in proline synthetic pathway were all reduced in cells of G2 and G3 (Fig. 2e). The contents of glycine and lysine in cells were also increased as a response to FAP stress, and then returned to low level with the adaptation to FAP (Fig. 2e). In our results, myo-inositol was also significantly affected by FAP. As shown in Fig. 2e, the cells of G1 were characterized by lower levels of myo-inositol compared to cells of G0 in FAP-free medium. In *S. cerevisiae*, the gene *PIS1* encodes the phosphatidylinositol synthase to catalyze the *de novo* synthesis of PI from myo-inositol and CDP-diacylglycerol. When yeast cells were treated by FAP, the transcriptional level of gene *PIS1* was distinctly elevated [26]. Thus, more myo-inositol would be applied for PI synthesis under FAP stress, which might reduce the intracellular accumulation of myo-inositol as observed in cells of G1. With the adaptation to FAP, myo-inositol content returned to high level.

**Fig. 3:**
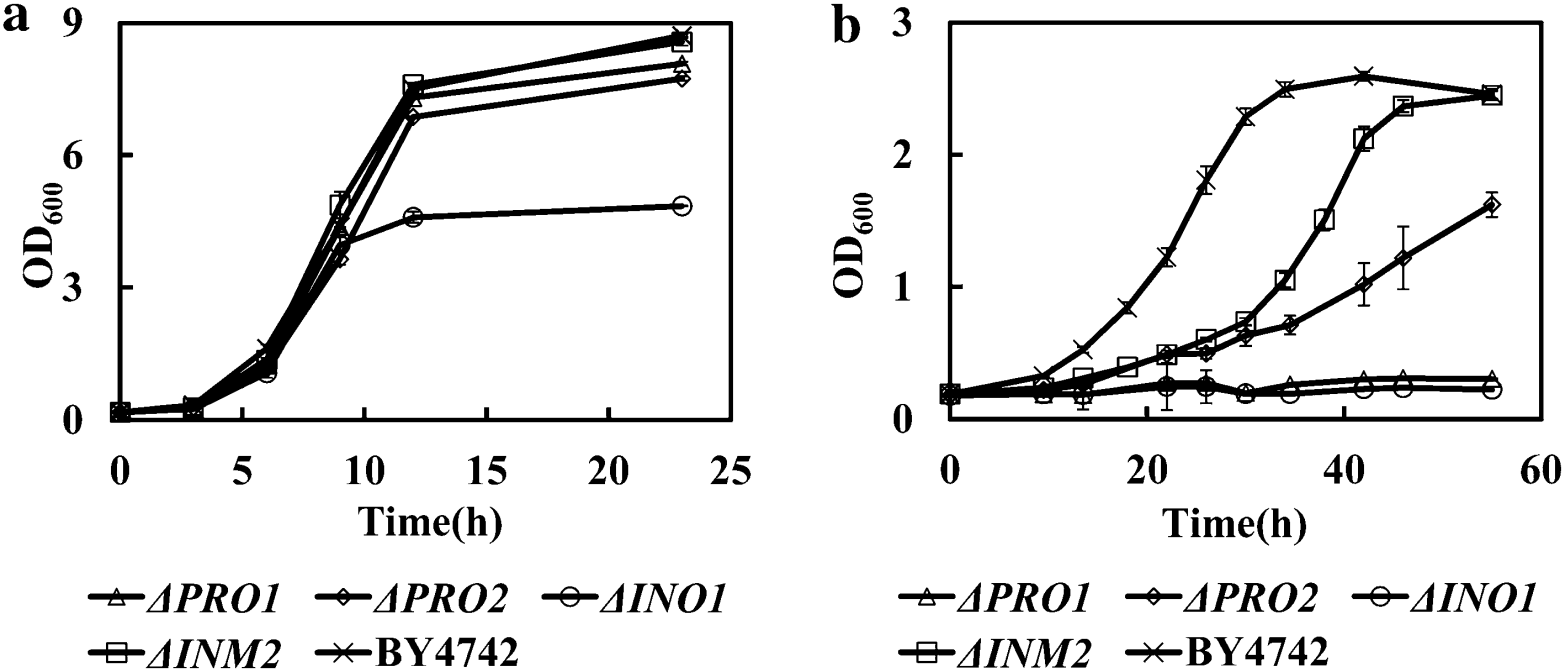
The effect of disturbing proline or myo-inositol biosynthesis on cell growth. **a** Cell growth of strain BY4742, *ΔPRO1*, *ΔPRO2*, *ΔINO1* and *ΔINM2* in YPD medium in the absence of multiple inhibitors. **b** Cell growth of strain BY4742, *ΔPRO1*, *ΔPRO2*, *ΔINO1* and *ΔINM2* in YPD medium in the presence of 1.0 g/L furfural, 4.0 g/L acetic acid and 0.4 g/L phenol. Results are the mean of duplicate experiments and *error bars* indicate SD

According to the above results, we predicted that the synthesis of proline, myo-inositol, glycine and lysine might be important for strain tolerance to FAP. The growth phenotypes of the mutants deficient in the synthesis of proline (*ΔPRO1* and *ΔPRO2*), glycine (*ΔGLY1* and *ΔSHM1*), lysine (*ΔLYS1)* and myo-inositol (*ΔINO1* and *ΔINM2*) were investigated to identify whether genetically disturbing the synthesis of these metabolites could affect cell tolerance to FAP. As shown in Additional file 3: Figure S2, the genetic perturbation of glycine or lysine biosynthesis through disrupting relevant gene *GLY1*, *SHM1* or *LYS1* does not affect cell growth no matter with or without combined inhibitors. However, the four mutants involved in proline synthesis and myo-inositol synthesis all exhibited increased sensitivity to FAP with enlarged lag phase and fermentation time compared to the parental strain (Fig. 3b). In the absence of FAP, the disruption of gene *PRO1*, *PRO2* and *INM2* had no effect on cell growth (Fig. 3a). The deletion of gene *INO1* triggered a lower final biomass but the fermentation time was slightly affected (Fig. 3a). These results proved the important role of proline and myo-inositol biosynthesis in maintaining strain growth ability under FAP stress. Accordingly, a schematic view of the targets which might be responsible for strain tolerance to FAP was proposed (Additional file 4: Figure S3).

### Effects of proline or myo-inositol supplementation on tolerance of *S. cerevisiae* to FAP

**Fig. 4:**
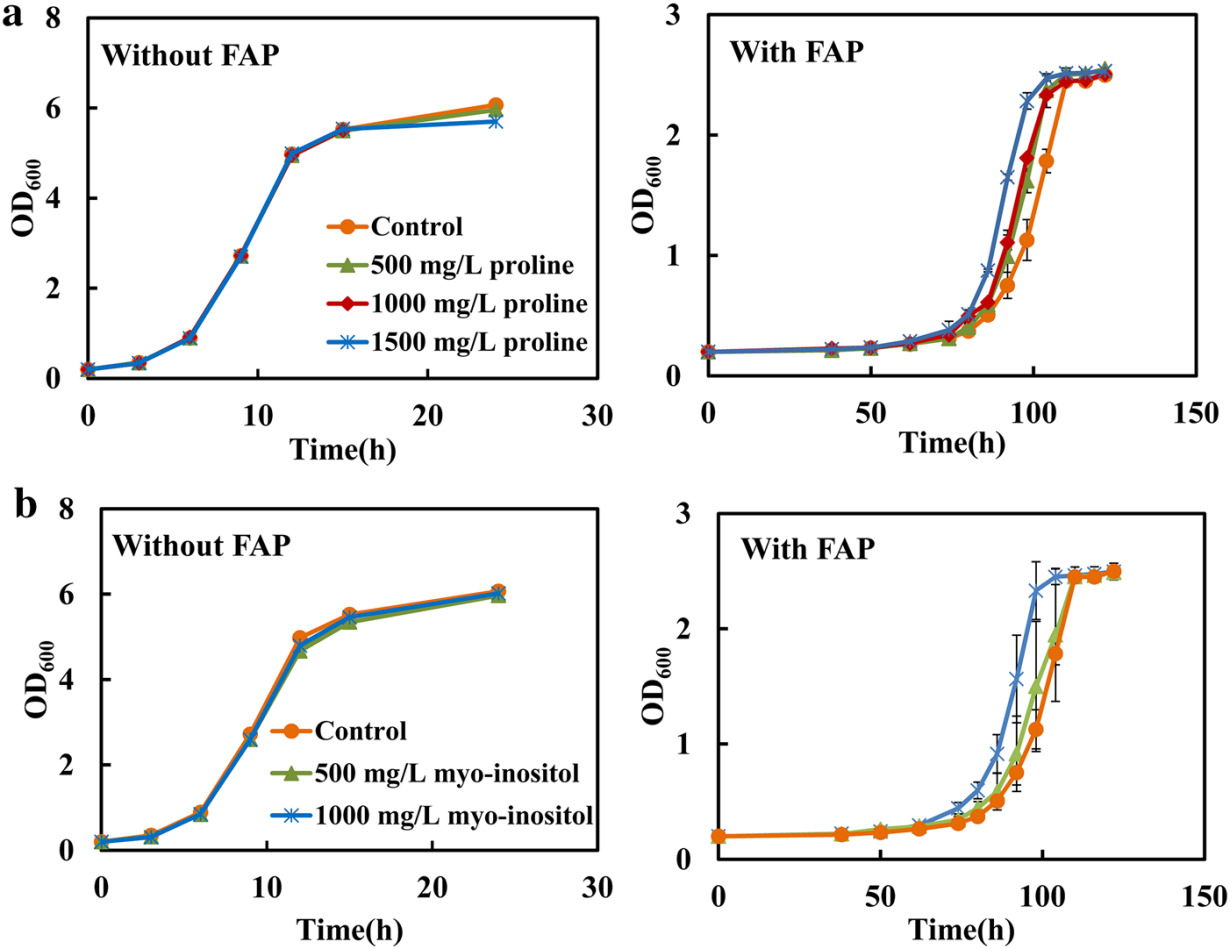
The effect of supplementation of proline or myo-inositol on cell tolerance against FAP stress. **a** The strain BY4742/pRS426 was cultivated in SC-Ura medium supplemented with 0, 500, 1000 and 1500 mg/L proline in the absence and presence of 0.8 g/L furfural, 3.0 g/L acetic acid and 0.3 g/L phenol. **b** The strain BY4742/pRS426 was cultivated in SC-Ura medium supplemented with 0, 500 and 1000 mg/L myo-inositol. Results are the mean of duplicate experiments and *error bars* indicate SD

Considering the above results, we speculated that strain tolerance to FAP could be improved through increasing the availability of proline or myo-inositol either by external addition or enhancing their intracellular synthesis. In the following study, the effect of proline or myo-inositol supplementation on the tolerance of strain BY4742/pRS426 to FAP was first tested. As shown in Fig. 4a, the addition of proline exerted a protective effect on cell growth under FAP stress. When extra 500 or 1000 mg/L proline was added, the moderate growth advantage was observed under FAP stress (Fig. 4a). Supplementation of 1500 mg/L proline further shortened the lag phase and fermentation time, and thus improved strain tolerance against FAP stress (Fig. 4a). For the effect of myo-inositol, the addition of extra 500 mg/L myo-inositol slightly increased strain growth ability under FAP stress, while an obvious growth advantage was observed when 1000 mg/L myo-inositol was added (Fig. 4b). In the FAP-free medium, neither proline nor myo-inositol addition has the positive effect on cell growth phenotypes (Fig. 4), indicating that the growth advantage appeared under FAP stress is not due to the minimal dose requirement for proline and myo-inositol. In addition, we found that the combination of proline and myo-inositol supplementation could also induce a moderate growth improvement under FAP stress compared to the single addition of proline or myo-inositol (Additional file 5: Figure S4). These results demonstrated that increasing proline and myo-inositol by exogenous addition contributed to the enhanced FAP tolerance in *S. cerevisiae*.

### Effects of enhancing proline synthesis on tolerance of *S. cerevisiae* to FAP

**Fig. 5:**
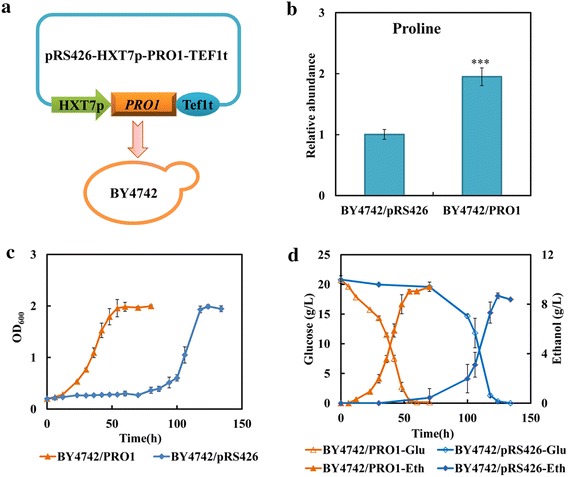
Enhancing proline synthesis improves strain tolerance to FAP. **a** The schematic of overexpressing *PRO1* to increase proline synthesis. **b** The effect of *PRO1* overexpression on the relative abundance of proline under FAP stress. **c** The effect of *PRO1* overexpression on the growth behaviors under FAP stress. **d** The effect of *PRO1* overexpression on the glucose consumption and ethanol production under FAP stress. The strain BY4742/pRS426 and recombinant strain BY4742/PRO1 were cultivated in SC-Ura medium in the presence of 0.8 g/L furfural, 3.0 g/L acetic acid and 0.3 g/L phenol. Results are the mean of duplicate experiments and *error bars* indicate SD

Although exogenously added proline or myo-inositol could alter yeast tolerance against combined inhibitors, this exogenous addition is not desirable for large-scale fermentations due to the additional costs. Therefore, to facilitate the enhanced tolerance without exogenous addition, we sought to genetically modify the strain by altering expression levels of the enzymes involved in proline or myo-inositol biosynthesis pathway to increase their intracellular synthesis. In *S. cerevisiae*, the main proline synthetic pathway from glutamate consisted of three enzymes: γ-glutamyl kinase (GK, the *PRO1* gene product), γ-glutamyl phosphate reductase (encoded by *PRO2*), and Δ^1^-pyrroline-5-carboxylate reductase (encoded by *PRO3*). The *PRO1* gene was first overexpressed to test its effect on strain tolerance against FAP stress (Fig. 5a). As shown in Fig. 5b, the overexpression of gene *PRO1* triggered approximate twofold higher intracellular proline in the recombinant strain (BY4742/PRO1) than that in the control strain (BY4742/pRS426). In FAP-free medium, the recombinant strain BY4742/PRO1 exhibited the similar fermentation pattern with the control strain (Additional file 6: Figure S5). When exposed to FAP condition, a great growth advantage was observed in strain BY4742/PRO1 compared to strain BY4742/pRS426 (Fig. 5c). The lag phase was significantly shortened due to the overexpression of *PRO1* gene (Fig. 5c). At about 54 h, the glucose could be depleted totally by the strain BY4742/PRO1, while the control strain was still in lag phase (Fig. 5c, d). The ethanol production rate was in parallel with the glucose consumption rate and ethanol yield was slightly affected (Fig. 5d). As mentioned above, the duration of the lag phase has been well established to evaluate the strain tolerance to inhibitors [20, 21]. The significantly shortened lag phase and increased glucose consumption rate of strain BY4742/PRO1 indicated that the enhancement of proline synthesis through overexpressing gene *PRO1* could successfully improve strain tolerance against FAP stress. However, the recombinant strain with the overexpression of gene *PRO2* did not exhibit the enhanced tolerance to FAP (Additional file 7: Figure S6). In the proline synthesis of *S. cerevisiae*, GK activity (encoded by *PRO1*) is sensitive to feedback inhibition by proline, and GK has been proved to be the rate-limiting and key regulatory enzyme that controls intracellular proline biosynthesis [27, 28]. Therefore, the gene *PRO1* was generally modified to regulate the proline level in *S. cerevisiae*. Through overexpressing the wild *PRO1* gene or expressing the mutant *PRO1* gene encoding the D154N mutant GK, which is less sensitive to proline feedback inhibition, increased proline accumulation *in S. cerevisiae* can be implemented and the enhanced tolerance was observed under freezing, air-drying, ethanol and high-sucrose stresses [29–32]. In our study, the overexpression of gene *PRO1* could also enhance proline accumulation and successfully improved strain tolerance to FAP. Correspondingly, the overexpression of *PRO2* gene might not play an active role in regulating intracellular proline synthesis to affect strain tolerance against FAP due to the strictly regulated role of GK on proline synthesis. The *PRO1* gene would be an alternative target for further improving strain tolerance against lignocellulose-derived inhibitors.

**Fig. 6:**
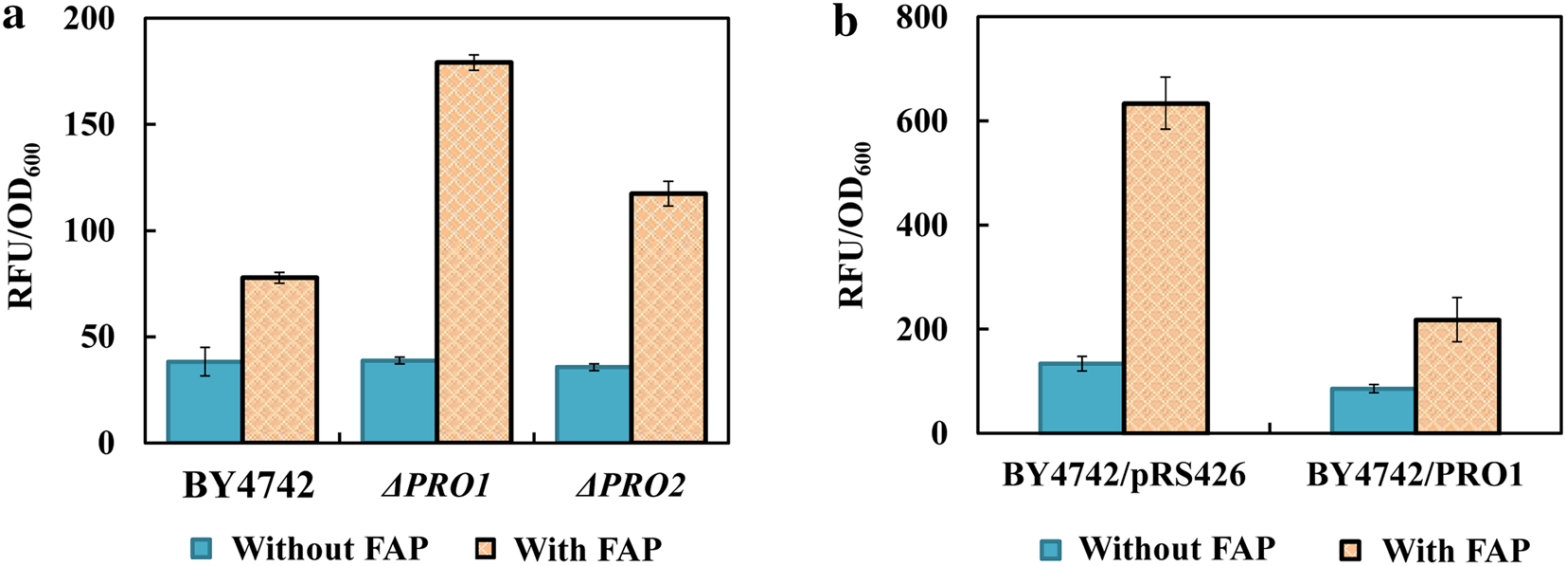
The proline synthesis affects the intracellular ROS level under FAP stress. **a** The effect of deleting genes involved in proline synthesis on intracellular ROS level. The strain BY4742, *ΔPRO1* and *ΔPRO2* were cultivated in YPD medium in the presence and absence of 1.0 g/L furfural, 4.0 g/L acetic acid and 0.4 g/L phenol. **b** The effect of *PRO1* overexpression on intracellular ROS level. The strain BY4742/pRS426 and strain BY4742/PRO1 were cultivated in SC-Ura medium in the presence of 0.8 g/L furfural, 3.0 g/L acetic acid and 0.3 g/L phenol. Relative fluorescence unit (RFU) per OD_600_ of cells was measured both in the absence and presence of combined inhibitors. Results are expressed as mean ± standard error of the mean (n = 4)

Proline involves in many important intracellular functions, such as maintaining protein and membrane stabilization, lowering the Tm of DNA, and scavenging of reactive oxygen species (ROS) [33]. Allen demonstrated that furfural induced ROS accumulation in *S. cerevisiae*, resulting in damage to mitochondria and vacuole membranes, the actin cytoskeleton and nuclear chromatin [34]. Acetic acid could induce a programmed cell death process with an apoptotic phenotype, which was related to the intracellular ROS level [35, 36]. As shown in Fig. 6, ROS accumulation was detected both in the parental strain and recombinant strains after the addition of FAP, reflecting that the multiple inhibitors might cause oxidative stress in yeast strain. As an antioxidant, proline has the ability to scavenge intracellular ROS and thereby suppresses ROS-mediated apoptosis [37]. Under FAP-free condition, the ROS levels in *PRO1*Δ and *PRO2*Δ were similar with their control strain BY4742. However, when cells were challenged by FAP, the interruption of proline biosynthesis by deleting gene *PRO1* or *PRO2* resulted in much higher ROS level accumulated in cells compared to the control strain BY4742 (Fig. 6a). On the contrary, the enhancement of proline biosynthesis by overexpressing gene *PRO1* largely mitigated the ROS accumulation in vivo (Fig. 6b). The ROS level in strain BY4742/PRO1 is only one-third of that in the control strain BY4742/pRS426 under FAP stress. Therefore, we predicted that one potential role of proline under FAP stress is serving as an ROS scavenger to protect cells from oxidative damage.

### Effects of enhancing myo-inositol synthesis on tolerance of *S. cerevisiae* to FAP

**Fig. 7:**
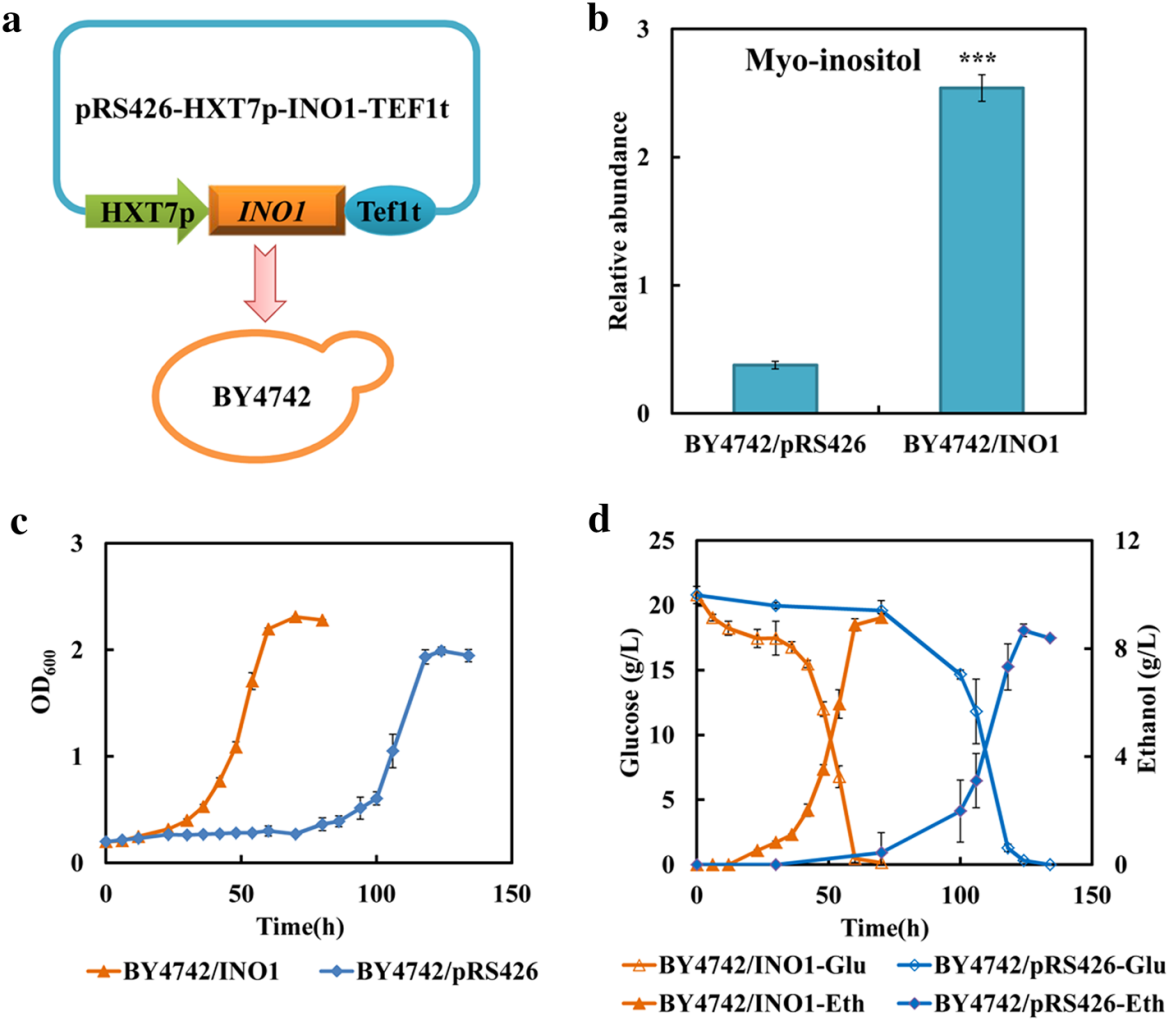
Enhancing myo-inositol synthesis improves strain tolerance to FAP. **a** The schematic of overexpressing *INO1* to up-regulate myo-inositol synthesis. **b** The effect of *INO1* overexpression on the relative abundance of myo-inositol under FAP stress. **c** The effect of *INO1* overexpression on the growth behaviors under FAP stress. **d** The effect of *INO1* overexpression on the glucose consumption and ethanol production under FAP stress. The strain BY4742/pRS426 and BY4742/INO1 were cultivated in SC-Ura medium in the presence of 0.8 g/L furfural, 3.0 g/L acetic acid and 0.3 g/L phenol. Results are the mean of duplicate experiments and *error bars* indicate SD

Myo-inositol is a precursor for many inositol-containing compounds and implicated in various physiological and biochemical processes such as membrane phospholipid synthesis, nuclear processes and alcohol stress [17, 38, 39]. In *S. cerevisiae*, myo-inositol is produced from glucose-6-phosphate via the reactions catalyzed by the *INO1*-encoded inositol-3-phosphate synthase and *INM1*-encoded inositol monophosphatase, and then participated in the biosynthesis of phosphatidylinositol (PI). The reaction catalyzed by *INO1*-encoded enzyme is known to be the key step in the synthesis of inositol-containing phospholipids. To engineer the strain with enhanced myo-inositol synthesis, the key gene *INO1* was overexpressed (Fig. 7a). The intracellular myo-inositol level of recombinant strain BY4742/INO1 was eightfold higher than that of the strain BY4742/pRS426 (Fig. 7b). The slight difference was observed between these two strains when the fermentation was performed in SC-Ura medium without FAP (Additional file 8: Figure S7). However, in the presence of FAP, the overexpression of gene *INO1* significantly reduced the lag phase and the strain BY4742/INO1 was more tolerant to inhibitors than the control strain (Fig. 7c). As illustrated in Fig. 7c, d, the strain BY4742/INO1 could grow into the stationary phase and exhaust the glucose totally at about 60 h, while the strain BY4742/pRS26 was still in the lag phase. The ethanol production rate was also in parallel with the glucose utilization, and the final ethanol production was slightly affected (Fig. 7d). Meanwhile, the overexpression of gene *INM2* could also improve strain ability to resist FAP stress (Additional file 7: Figure S6). These results further indicated that myo-inositol metabolism was one of the targets facilitating strain tolerance to FAP. Increasing myo-inositol by the overexpression of genes involved in myo-inositol biosynthesis could improve strain tolerance against multiple inhibitors. However, the role of myo-inositol in protecting cells against FAP was still unclear. As a direct precursor of PI synthesis, intracellular myo-inositol level may affect the lipid composition and membrane integrity [39]. Yang et al. [26] has reported that combined inhibitors interrupted the membrane integrity and permeability, and the increase of PCs and PIs with long fatty acyl chains might be an important compensatory mechanism for the increase of plasma membrane permeability and fluidity when subjected to combined inhibitors. Therefore, it was most likely that myo-inositol acted on cell membrane to play its protective role in resisting tolerance.

## Conclusions

In this study, through a comparative metabolomic analysis, proline and myo-inositol syntheses were postulated to be the key elements in yeast tolerance to the mixture of furfural, acetic acid and phenol. The deletion of genes involved in proline or myo-inositol synthetic pathway increased the sensitivity of yeast to multiple inhibitors. On the contrary, the external addition of proline and myo-inositol or enhancing their intracellular synthesis by overexpressing key genes involved in proline or myo-inositol synthetic pathway successfully conferred the yeast strains enhanced tolerance to FAP by significantly reducing the lag phase time. Furthermore, ROS determination indicated that the enhancement of proline synthesis could remove the extra intracellular ROS induced by FAP. These findings provide valuable insights into the engineering of robust microbes for efficient cellulosic ethanol production.

## Methods

### Microbial strains and media

Microbial strains used in this study are listed in Table 1. Yeast strains were cultivated in liquid synthetic complete medium without uracil (SC-Ura; 20 g/L glucose, 6.7 g/L yeast nitrogen base without amino acids, 2 g/L amino acid powder mixture lacking histidine, tryptophane, leucine and uracil, 20 mg/L histidine, 20 mg/L tryptophane and 100 mg/L leucine), or in YPD medium (20 g/L glucose, 20 g/L peptone and 10 mg/L yeast extract). *Escherichia coli* DH5α was grown in Luria–Bertani medium (10 g/L peptone, 5 g/L yeast extract and 5 g/L sodium chloride) containing 100 mg/L ampicillin.

**Table 1:** Yeast strains and plasmids used in this study

Yeast strains or plasmids	Description	References
*S. cerevisiae* strains		
BY4741	*MATa HIS3 LEU2 MET15 URA3*	Research genetics
BY4742	*MATα HIS3 LEU2 LYS2 URA3*	Research genetics
BY4742-13659 (*ΔPRO1*)	Isogenic to BY4742, except *PRO1::KAN*	Research genetics
BY4742-11620 (*ΔPRO2*)	Isogenic to BY4742, except *PRO2::KAN*	Research genetics
BY4742-11272 (*ΔINO1*)	Isogenic to BY4742, except *INO1::KAN*	Research genetics
BY4742-13646 (*ΔINM2*)	Isogenic to BY4742, except *INM2::KAN*	Research genetics
BY4742-10287 (*ΔGLY1*)	Isogenic to BY4742, except *GLY1::KAN*	Research genetics
BY4742-15969 (*ΔLYS1*)	Isogenic to BY4742, except *LYS1::KAN*	Research genetics
BY4742-13403 (*ΔSHM1*)	Isogenic to BY4742, except *SHM1::KAN*	Research genetics
BY4742/PRO1	BY4742 (pRS426-HXT7p-PRO1-TEF1t)	This study
BY4742/INO1	BY4742 (pRS426-HXT7p-INO1-TEF1t)	This study
BY4742/pRS426	BY4742 (pRS426)	This study
BY4742/PRO2	BY4742 (pRS426-HXT7p-PRO2-TEF1t)	This study
BY4742/INM2	BY4742 (pRS426-HXT7p-INM2-TEF1t)	This study
Plasmid		
pRS426	*URA3*, no expression (control plasmid)	[45]
pRS426-HXT7p-PRO1-TEF1t	*URA3*, pRS426 with *PRO1* inserted between HXT7 promoter and TEF1 terminator	This study
pRS426-HXT7p-INO1-TEF1t	*URA3*, pRS426 with *INO1* inserted between HXT7 promoter and TEF1 terminator	This study
pRS426-HXT7p-PRO2-TEF1t	*URA3*, pRS426 with *PRO2* inserted between HXT7 promoter and TEF1 terminator	This study
pRS426-HXT7p-INM2-TEF1t	*URA3*, pRS426 with *INM2* inserted between HXT7 promoter and TEF1 terminator	This study

### Construction of plasmids and yeast transformation

Plasmids constructed in this study are listed in Table 1 and Additional file 9: Table S2. The *HXT7* promoter and *TEF1* terminator were amplified from the genomic DNA of *S. cerevisiae* S288C. The *HXT7* promoter was digested with BamHI and EcoRI and subsequently ligated into pRS426 to generate plasmid pRS426-HXT7p. After digestion with SalI and XhoI, the *TEF1* terminator was inserted into pRS426-HXT7p to obtain plasmid pRS426-HXT7p-TEF1t. *PRO1*, *INO1* and *INM2* open reading frames were amplified from the genomic DNA of strain BY4742. The PCR products were digested with EcoRI and SalI, and cloned into pRS426-HXT7p-TEF1t to yield plasmid pRS426-HXT7p-PRO1-TEF1t, pRS426-HXT7p-INO1-TEF1t, and pRS426-HXT7p-INM2-TEF1t, respectively. The *PRO2* gene was also amplified from the same genomic DNA. Subsequently, the PRO2-TEF1t fragment was generated by overlapping PCR and cloned into the EcoRI and XhoI sites of pRS426-HXT7p to obtain plasmid pRS426-HXT7p-PRO2-TEF1t.

Yeast transformation was performed using the lithium acetate/single-stranded carrier DNA/PEG method as described previously [40, 41]. Transformants were selected on SC-Ura plate.

### Growth assays

An adaptation experiment was first carried out in yeast strain BY4741 (Research Genetics Inc., Huntsville, AL, USA) as the schematic shown in Fig. 2a. A single colony of strain was first pre-cultivated in YPD medium at 30 °C for 12 h. Then, seed cultures with an initial optical density (OD_600_) of 1.0 were transferred into a 5-L fermenter (1.5BG-4-3000, BXBIO, Shanghai, China) containing 3 L YPD medium with or without 1.3 g/L furfural, 5.3 g/L acetic acid and 0.5 g/L phenol (100 % FAP). The fermenter was maintained at 30 °C and 300 rpm anaerobically till cultures reached stationary phase. Then appropriate amounts of cell pellets in FAP-containing medium were collected by centrifugation at 4000×*g* and transferred into the fresh YPD medium containing same concentration of inhibitors at an initial OD_600_ of 1.0. Two transfers were carried out. These three cultivation process was referred as “G1”, “G2” and “G3”, respectively, according to their transfer sequence. The cultivation process in FAP-free medium was referred as “G0”. Yeast cells were harvested at the lag phase of each cultivation process during the adaptation to FAP for comprehensive metabolomic analysis.

The yeast transformants were cultivated in SC-Ura medium for 20 h at 30 °C. Cells were collected by centrifugation at 3000×*g* for 10 min and inoculated into 100 mL fermentation medium (SC-Ura) supplemented with or without 60 % FAP (0.8 g/L furfural, 3.0 g/L acetic acid and 0.3 g/L phenol). The fermentation was performed at 30 °C and 150 rpm. The flask was capped with rubber stopper with a syringe needle. The initial OD_600_ was adjusted to 0.2.

To test the effect of proline or myo-inositol supplementation on cell FAP sensitivity, overnight seed cultures of the control strain (BY4742/pRS426) were incubated in SC-Ura medium supplemented with different concentration of proline or myo-inositol in the absence and presence of 60 % FAP. The cultivation condition was the same with the description above.

The related gene deletions (Open Biosystems, Huntsville, AL) were plated on the YPD-agar plate with neomycin analogue G418 (200 μg/mL). Then a single clone of the mutants was inoculated in YPD medium for activation twice. Subsequently, the growth of the mutants was tested in 100 mL YPD medium with and without 80 % FAP (1.0 g/L furfural, 4.0 g/L acetic acid and 0.4 g/L phenol) at 30 °C and 150 rpm, and the strain BY4742 was used as a control.

### Analytical methods

The concentration of glucose and ethanol was analyzed by Waters HPLC (high-performance liquid chromatograph) system equipped with an Aminex HPX-87H ion-exchange column (Bio-Rad, Hercules, CA, USA) and a Waters 2414 refractive index detector.

### Metabolite extraction and analysis

Quenching, metabolite extraction and derivatization were performed according to methods described previously [23]. The samples were analyzed by an Agilent 6890 gas chromatograph coupled with Waters time-of-flight mass spectrometry (GC–TOF-MS), which was equipped with a DB-5 fused-silica capillary column (30 m × 0.25 mm i.d., film thickness 0.25 µm, J&W Scientific, Folsom, CA). The instrument method was also the same with the previous report [23]. Metabolite identification and quantification were performed using Masslynx software (Waters Corp., USA). For the metabolite identification, the mass spectra of each peak were matched with the National Institute of Standards and Technology mass spectral library (NIST 2010). The area of each acquired peak was then normalized to that of internal standard in the same chromatogram for further data processing.

### Multivariate statistical analysis

#### Partial least squares-discriminant analysis (PLS-DA)

PLS-DA was performed using SIMCA-P 11.5 Demo software after mean-centering and pareto-scaling the normalized metabolite datasets. PLS-DA score plot, which was shown of the two first predictive components (t[1]P and t[2]P) in this study, interpreted differences and similarities among the data samples. PLS-DA variable loading plot was used to extract the metabolites that contributed the most to the classification.

#### Minimum redundancy maximum relevance (mRMR)

The method of mRMR was originally developed for analyzing the microarray data [42]. In this study, using the statistics toolbox of Matlab (The MathWorks, R2008a), the analysis of mRMR ranked the metabolites according to their relevance to the class of samples concerned, and also takes the redundancy of metabolites into account. Those metabolites, which have the best trade-off between the maximum relevance to the sample class and the minimum redundancy, were considered as “good” biomarkers. Both the relevance and redundancy were quantified by the mutual information.

#### Metabolic pathway analysis

Metabolic pathway analysis combines the result from powerful pathway enrichment analysis and pathway topology analysis to identify the most relevant metabolic pathways, which was performed with MetPA, a part of MetaboAnalyst 2.0 (Canada) (http://www.metaboanalyst.ca/MetaboAnalyst/) [43]. MetPA uses high-quality KEGG metabolic pathways as the back-end knowledge [43, 44]. In this study, the data were first mean-centered and pareto-scaled, and then we selected the ‘*Saccharomyces cerevisiae*’ library and used the default ‘Global Test’ and ‘Relative Betweenness Centrality’ for pathway analysis.

### ROS assays

Cell pellets were washed with phosphate-buffered saline (PBS, pH 7.0) and resuspended in PBS at a final concentration of 10^7^ cells/mL. 10 µg of 2′,7′-dichlorofluorescein diacetate (DCF) (Sigma-35845) (using a 2.5 mg/mL stock dissolved in DMSO) was added to 1 mL of cell suspension. After incubation at 30 °C for 60 min, cells were washed twice and resuspended in 1 mL PBS. The relative fluorescence intensity was measured by a Cary Eclipse Spectrofluorimeter (Varian, Walnut Creek., USA) (excitation, 488 nm; emission, 525 nm). The OD_600_ value of the cell suspension was also measured.

## Additional files

**Additional file 1: Table S1.** Metabolites detected in samples of G0, G1, G2 and G3.
**Additional file 2: Figure S1.** Variations of citrate during the adaptation process to multiple inhibitors. The relative abundance was calculated by normalizing the peak area of citrate with internal standard (IS) in the same chromatogram. Results are expressed as mean ± standard error of the mean (n > 5).
**Additional file 3: Figure S2.** The growth behaviors of the strain BY4742, *ΔGLY1*, *ΔSHM1* and *ΔLYS1*. (a) The strains were cultivated in YPD medium in the absence of multiple inhibitors. (b) The strains were cultivated in YPD medium in the presence of 1.0 g/L furfural, 4.0 g/L acetic acid and 0.4 g/L phenol. Results are the mean of duplicate experiments and error bars indicate SD.
**Additional file 4: Figure S3.**The predicted targets important for strain resisting multiple inhibitors (furfural, acetic acid and phenol) in *S. cerevisiae.*

**Additional file 5: Figure S4.** Effects of combination of proline and myo-inositol supplementation on cell tolerance against FAP stress. (A) The strain BY4742/pRS426 was cultivated in SC-Ura medium supplemented with 1000 mg/L myo-inositol and 1500 mg/L proline; (B) The strain BY4742/pRS426 was cultivated in SC-Ura medium supplemented with 1000 mg/L myo-inositol, 1500 mg/L proline or the combination of 1000 mg/L myo-inositol and 1500 mg/L proline in the presence of 0.8 g/L furfural, 3.0 g/L acetic acid and 0.3 g/L phenol. Results are the mean of duplicate experiments and error bars indicate SD.
**Additional file 6: Figure S5.** The growth profiles, glucose consumption and ethanol production of the strain BY4742/PRO1 and the control strain BY4742/pRS426 in SC-Ura medium in the absence of multiple inhibitors. Results are the mean of duplicate experiments and error bars indicate SD.
**Additional file 7: Figure S6.** Effects of overexpression of gene *PRO2* or *INM2* in proline or myo-inositol biosynthetic pathway on cell growth. The recombinant strain BY4742/PRO2 and strain BY4742/INM2 were cultivated in SC-Ura medium in the presence and absence of 0.8 g/L furfural, 3.0 g/L acetic acid and 0.3 g/L phenol. The strain BY4742/pRS426 was performed as the control. Results are the mean of duplicate experiments and error bars indicate SD.
**Additional file 8: Figure S7.** The growth profiles, glucose consumption and ethanol production of the recombinant strain BY4742/INO1 and its control strain BY4742/pRS426 in SC-Ura medium in the absence of multiple inhibitors. Results are the mean of duplicate experiments and error bars indicate SD.
**Additional file 9: Table S2.** Primers used in this study with endonuclease restriction sites underlined and italicized as necessary.

## Abbreviations

PLS-DA: partial least squares-discriminant analysis
mRMR: minimal redundancy maximal relevance
FAP: furfural, acetic acid and phenol
ROS: reactive oxygen species
PI: phosphatidylinositol
SSA: succinate semialdehyde
Glycerol-3-P: glycerol-3-phosphate
GABA: γ-aminobutyric acid
DHAP: dihydroxyacetone phosphate
