# Supplementary material for: Increasing proline and myo-inositol improves tolerance of Saccharomyces cerevisiae to the mixture of multiple lignocellulose-derived inhibitors
Source: Biotechnol Biofuels. 2015 Sep 15;8:142. doi: 10.1186/s13068-015-0329-5 (PMC4570682; doi:10.1186/s13068-015-0329-5)
Supplement: Supplementary file 1 — Additional file 1: Table S1. Metabolites detected in samples of G0, G1, G2 and G3. [file 13068_2015_329_MOESM1_ESM.pdf]

Table S1 Relative level of metabolites detected in samples of G0, G1, G2 and G3. The value was calculated by normalizing the peak area of each metabolite to internal standard in the same chromatogram. Results are shown as mean ± standard error of the mean (n≥5).

ID	Metabolites	G0	G1	G2	G3
**Sugars**					
M1	Glucose	0.266±0.012	1.191±0.188	0.918±0.088	0.677±0.129
M2	Trehalose	0.003±0.001	0.000±0.000	0.048±0.005	0.017±0.004
M3	Ribose	0.071±0.010	0.007±0.001	0.042±0.008	0.076±0.011
M4	Fructose	0.022±0.005	0.027±0.002	0.028±0.007	0.048±0.006
M5	Galactose	0.088±0.013	0.208±0.009	0.211±0.013	0.329±0.012
M6	Allose	0.082±0.015	0.155±0.011	0.123±0.021	0.077±0.017
M7	Mannose	0.087±0.011	0.103±0.070	0.101±0.029	0.159±0.012
**Amino acids**					
M8	Valine	0.212±0.037	0.246±0.017	0.155±0.011	0.257±0.021
M9	Alanine	1.376±0.286	10.771±1.829	2.484±0.477	1.658±0.218
M10	Leucine	0.027±0.006	0.012±0.001	0.015±0.002	0.034±0.003
M11	α-aminobutyric acid	0.018±0.003	0.027±0.002	0.030±0.003	0.037±0.004
M12	Isoleucine	0.102±0.029	0.107±0.009	0.060±0.015	0.148±0.019
M13	Proline	0.729±0.136	1.362±0.156	0.219±0.025	0.196±0.013
M14	Glycine	3.960±0.508	5.523±0.139	3.227±0.198	4.481±0.135
M15	Serine	0.371±0.059	0.692±0.080	0.759±0.060	0.991±0.076
M16	Threonine	0.255±0.043	0.327±0.020	0.202±0.013	0.338±0.032
M17	Aminomalonic acid	0.041±0.005	0.169±0.020	0.049±0.007	0.052±0.011
M18	5-Oxo-proline	3.218±0.418	1.228±0.041	1.009±0.086	0.888±0.050
M19	Aspartate	1.829±0.347	0.656±0.139	0.288±0.051	0.754±0.119
M20	Hydroxyproline	0.078±0.013	0.110±0.007	0.012±0.003	0.007±0.002
M21	GABA	0.051±0.007	1.6860.065	0.171±0.017	0.127±0.013
M22	N-acetyl-glutamic acid	0.282±0.052	0.08±0.007	0.080±0.012	0.075±0.010
M23	Ornithine	8.852±1.632	6.502±0.746	1.219±0.055	1.078±0.104
M24	Glutamate	24.209±3.060	8.191±0.750	5.693±0.971	6.637±0.522
M25	Asparagine	0.045±0.012	0.046±0.007	0.029±0.005	0.042±0.007
M26	Glutamine	0.730±0.103	0.224±0.006	0.091±0.010	0.040±0.009
M27	Histidine	0.086±0.021	0.085±0.006	0.034±0.008	0.043±0.007
M28	Lysine	13.894±1.524	15.858±0.741	5.710±0.334	6.601±0.243
M29	Tyrosine	0.369±0.046	0.186±0.023	0.039±0.005	0.132±0.010
M30	Tryotophan	0.004±0.001	0.007±0.002	0.005±0.001	0.007±0.002
M31	α-Aminoadipic acid	0.243±0.044	0.359±0.017	0.087±0.006	0.135±0.011
**Polyamines**					
M32	Ethanolamine	0.233±0.030	0.331±0.014	0.382±0.016	0.339±0.015
M33	Hydroxylamine	0.011±0.002	0.015±0.001	0.016±0.003	0.014±0.002
M34	1,4-Butanediamine	0.129±0.013	0.151±0.009	0.407±0.042	0.455±0.027
M35	Cadaverine	0.066±0.018	0.060±0.010	0.012±0.002	0.012±0.003
M36	Dopamine	0.014±0.003	0.023±0.006	0.047±0.003	0.096±0.004
M37	Niacinamide	0.036±0.007	0.053±0.006	0.077±0.008	0.145±0.024
M38	Spermine	0.022±0.006	0.010±0.002	0.011±0.003	0.013±0.003
**Organic acids**					
M39	Pyruvic acid	0.018±0.004	0.022±0.001	0.017±0.001	0.021±0.002
M40	Lactic acid	0.262±0.042	0.319±0.072	0.276±0.048	0.287±0.048
M41	Propanoic acid	0.010±0.001	0.012±0.001	0.012±0.001	0.013±0.001

ID	Metabolites	G0	G1	G2	G3
M42	Glycolic acid	0.005 ±0.001	0.007 ±0.001	0.008 ±0.001	0.010 ±0.002
M43	2-Furoic acid	0.001 ±0.000	0.118 ±0.004	0.116 ±0.004	0.158 ±0.004
M44	Ethanedioic acid	1.118 ±0.126	1.214 ±0.159	1.236 ±0.091	1.371 ±0.079
M45	Succinic acid	0.349 ±0.062	1.173 ±0.122	0.551 ±0.049	0.647 ±0.064
M46	Fumaric acid	0.062 ±0.011	0.198 ±0.008	0.150 ±0.026	0.264 ±0.020
M47	Citric acid	1.891 ±0.327	3.317 ±0.533	2.336 ±0.253	1.285 ±0.046
M48	Octadecanoic acid	1.717 ±0.108	1.706 ±0.109	1.714 ±0.118	2.069 ±0.306
M49	9-Hexadecanoic acid	0.069 ±0.015	0.036 ±0.005	0.232 ±0.054	0.206 ±0.049
M50	Hexadecanoic acid	1.602 ±0.055	1.534 ±0.124	1.621 ±0.134	1.868 ±0.269
**Polyols**					
M51	2,3-Butanediol	0.014 ±0.003	0.133 ±0.016	0.081 ±0.006	0.099 ±0.009
M52	Glycerol	29.471 ±2.182	31.392 ±0.337	20.194 ±0.515	19.657 ±0.658
M53	Threitol	0.013 ±0.002	0.017 ±0.004	0.004 ±0.001	0.003 ±0.001
M54	Ribitol	0.009 ±0.003	0.020 ±0.001	0.016 ±0.002	0.031 ±0.004
M55	Arabinitol	0.018 ±0.003	0.040 ±0.003	0.061 ±0.008	0.081 ±0.009
M56	Mannitol	0.195 ±0.011	0.352 ±0.019	0.471 ±0.073	0.504 ±0.067
M57	Glucitol	0.794 ±0.103	1.098 ±0.159	1.537 ±0.040	1.173 ±0.133
M58	Myo-inositol	0.532 ±0.095	0.232 ±0.041	1.092 ±0.146	1.014 ±0.050
M59	Galactinol	0.180 ±0.032	0.115 ±0.008	0.119 ±0.012	0.225 ±0.006
**Others**					
M60	Phosphorate methyl ester	2.587 ±0.601	2.648 ±0.198	1.758 ±0.226	3.153 ±0.531
M61	Phosphorate	29.052 ±3.536	26.966 ±1.028	22.711 ±1.996	26.107 ±2.284
M62	Glycerol 3-phosphate	0.526 ±0.118	0.690 ±0.065	0.255 ±0.038	0.401 ±0.047
M63	Adenosine phosphate	0.022 ±0.005	0.029 ±0.006	0.016 ±0.003	0.043 ±0.004
M64	Urea	0.664 ±0.087	0.418 ±0.016	0.178 ±0.007	0.147 ±0.005
M65	Hypoxanthine	0.087 ±0.020	0.058 ±0.007	0.039 ±0.006	0.066 ±0.006
M66	Adenine	0.461 ±0.052	0.466 ±0.032	0.775 ±0.055	1.426 ±0.022
M67	Uridine	0.035 ±0.005	0.064 ±0.013	0.051 ±0.007	0.063 ±0.008
M68	Adenosine	0.012 ±0.003	0.011 ±0.003	0.017 ±0.004	0.047 ±0.006
M69	Uracil	0.149 ±0.024	0.009 ±0.002	0.013 ±0.002	0.028 ±0.004
M70	Uracil-4-carboxylic acid	0.504 ±0.117	1.204 ±0.044	0.365 ±0.052	0.884 ±0.051
