# Supplementary material for: Increasing proline and myo-inositol improves tolerance of Saccharomyces cerevisiae to the mixture of multiple lignocellulose-derived inhibitors
Source: Biotechnol Biofuels. 2015 Sep 15;8:142. doi: 10.1186/s13068-015-0329-5 (PMC4570682; doi:10.1186/s13068-015-0329-5)

Figure S1 Variations of citrate during the adaptation process to multiple inhibitors. The relative abundance was calculated by normalizing the peak area of citrate with internal standard (IS) in the same chromatogram. Results are expressed as mean ± standard error of the mean (n>5).

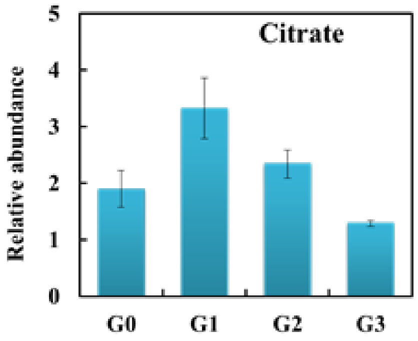

Supplement: Supplementary file 2 — Additional file 2: Figure S1. Variations of citrate during the adaptation process to multiple inhibitors. The relative abundance was calculated by normalizing the peak area of citrate with internal standard (IS) in the same chromatogram. Results are expressed as mean ± standard error of the mean (n > 5). [file 13068_2015_329_MOESM2_ESM.pdf]
