# Supplementary material for: Increasing proline and myo-inositol improves tolerance of Saccharomyces cerevisiae to the mixture of multiple lignocellulose-derived inhibitors
Source: Biotechnol Biofuels. 2015 Sep 15;8:142. doi: 10.1186/s13068-015-0329-5 (PMC4570682; doi:10.1186/s13068-015-0329-5)

Figure S2 The growth behaviors of the strain BY4742, *ΔGLY1*, *ΔSHM1* and *ΔLYS1*. (a) The strains were cultivated in YPD medium in the absence of multiple inhibitors. (b) The strains were cultivated in YPD medium in the presence of 1.0 g/L furfural, 4.0 g/L acetic acid and 0.4 g/L phenol. Results are the mean of duplicate experiments and error bars indicate s.d.

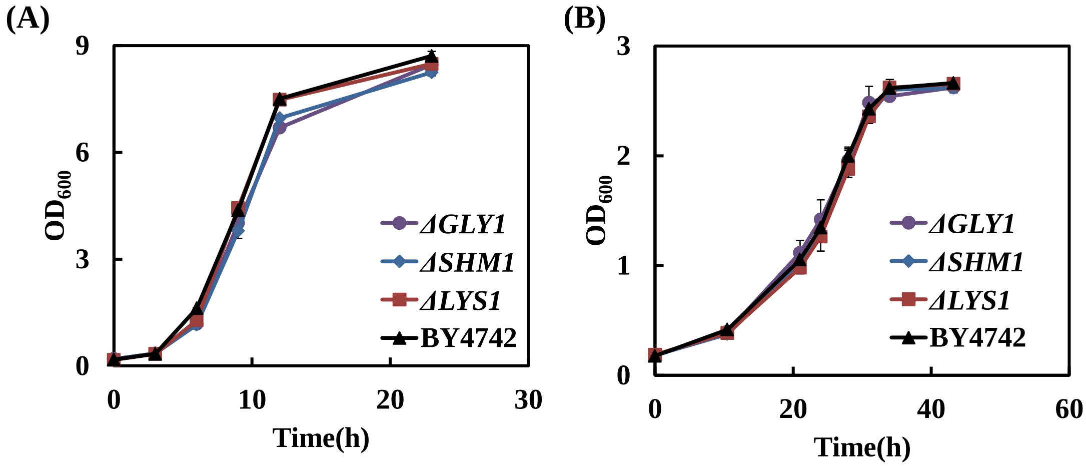

Supplement: Supplementary file 3 — Additional file 3: Figure S2. The growth behaviors of the strain BY4742, ΔGLY1, ΔSHM1 and ΔLYS1. (a) The strains were cultivated in YPD medium in the absence of multiple inhibitors. (b) The strains were cultivated in YPD medium in the presence of 1.0 g/L furfural, 4.0 g/L acetic acid and 0.4 g/L phenol. Results are the mean of duplicate experiments and error bars indicate SD. [file 13068_2015_329_MOESM3_ESM.pdf]
