# Supplementary material for: Increasing proline and myo-inositol improves tolerance of Saccharomyces cerevisiae to the mixture of multiple lignocellulose-derived inhibitors
Source: Biotechnol Biofuels. 2015 Sep 15;8:142. doi: 10.1186/s13068-015-0329-5 (PMC4570682; doi:10.1186/s13068-015-0329-5)

Figure S3 The predicted targets important for strain resisting multiple inhibitors (furfural, acetic acid and phenol) in *S. cerevisiae*.

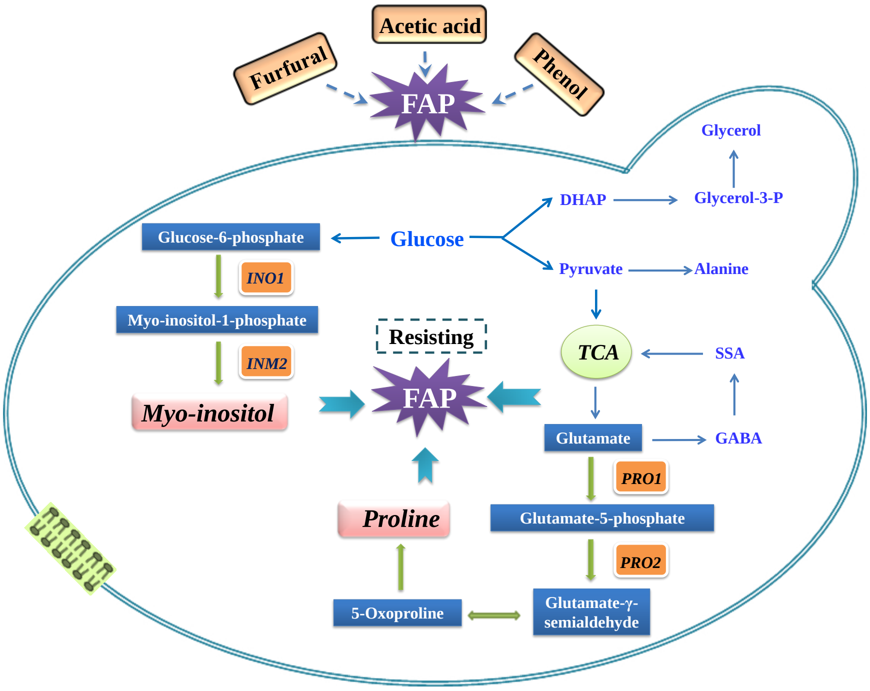

Supplement: Supplementary file 4 — Additional file 4: Figure S3.The predicted targets important for strain resisting multiple inhibitors (furfural, acetic acid and phenol) in S. cerevisiae. [file 13068_2015_329_MOESM4_ESM.pdf]
