# Supplementary material for: Increasing proline and myo-inositol improves tolerance of Saccharomyces cerevisiae to the mixture of multiple lignocellulose-derived inhibitors
Source: Biotechnol Biofuels. 2015 Sep 15;8:142. doi: 10.1186/s13068-015-0329-5 (PMC4570682; doi:10.1186/s13068-015-0329-5)

Figure S4 Effects of combination of proline and myo-inositol supplementation on cell tolerance against FAP stress. (A) The strain BY4742/pRS426 was cultivated in SC-Ura medium supplemented with 1000 mg/L myo-inositol and 1500 mg/L proline; (B) The strain BY4742/pRS426 was cultivated in SC-Ura medium supplemented with 1000 mg/L myo-inositol, 1500 mg/L proline or the combination of 1000 mg/L myo-inositol and 1500 mg/L proline in the presence of 0.8 g/L furfural, 3.0 g/L acetic acid and 0.3 g/L phenol. Results are the mean of duplicate experiments and error bars indicate s.d.

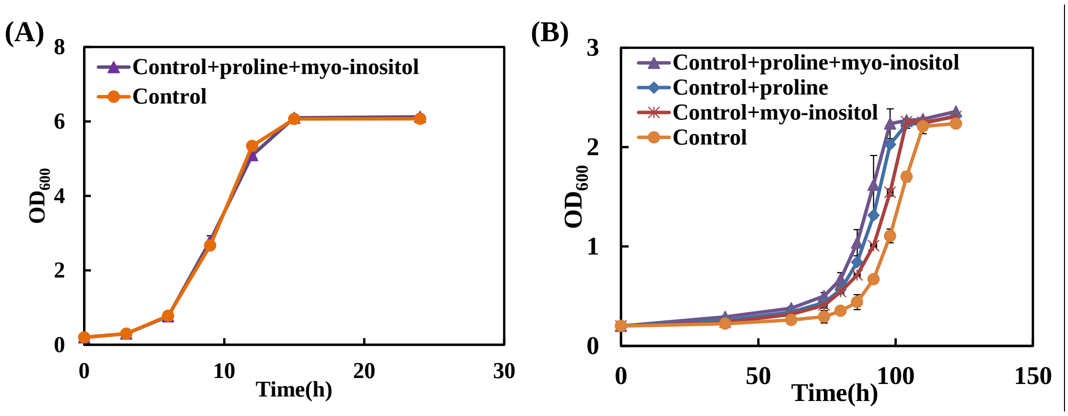

Supplement: Supplementary file 5 — Additional file 5: Figure S4. Effects of combination of proline and myo-inositol supplementation on cell tolerance against FAP stress. (A) The strain BY4742/pRS426 was cultivated in SC-Ura medium supplemented with 1000 mg/L myo-inositol and 1500 mg/L proline; (B) The strain BY4742/pRS426 was cultivated in SC-Ura medium supplemented with 1000 mg/L myo-inositol, 1500 mg/L proline or the combination of 1000 mg/L myo-inositol and 1500 mg/L proline in the presence of 0.8 g/L furfural, 3.0 g/L acetic acid and 0.3 g/L phenol. Results are the mean of duplicate experiments and error bars indicate SD. [file 13068_2015_329_MOESM5_ESM.pdf]
