# Supplementary material for: Increasing proline and myo-inositol improves tolerance of Saccharomyces cerevisiae to the mixture of multiple lignocellulose-derived inhibitors
Source: Biotechnol Biofuels. 2015 Sep 15;8:142. doi: 10.1186/s13068-015-0329-5 (PMC4570682; doi:10.1186/s13068-015-0329-5)

Figure S6 Effects of overexpression of gene *PRO2* or *INM2* in proline or myo-inositol biosynthetic pathway on cell growth. The recombinant strain BY4742/PRO2 and strain BY4742/INM2 were cultivated in SC-Ura medium in the presence and absence of 0.8 g/L furfural, 3.0 g/L acetic acid and 0.3 g/L phenol. The strain BY4742/pRS426 was performed as the control. Results are the mean of duplicate experiments and error bars indicate s.d.

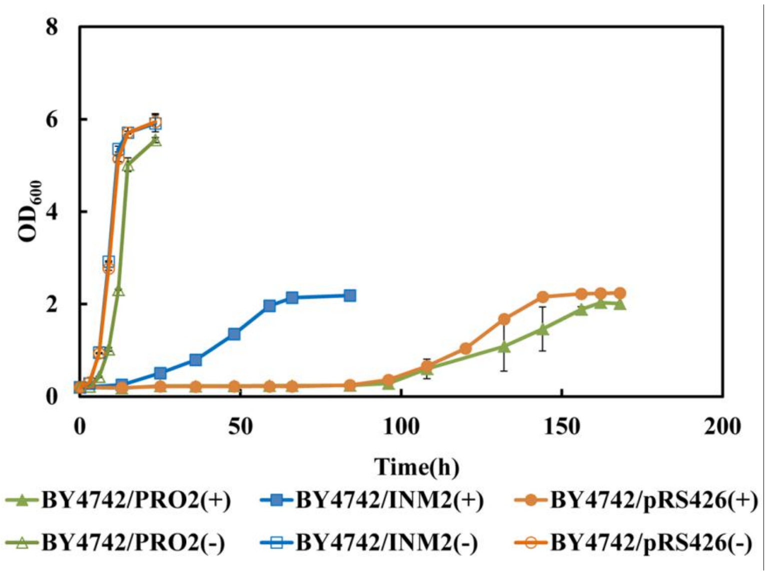

Supplement: Supplementary file 7 — Additional file 7: Figure S6. Effects of overexpression of gene PRO2 or INM2 in proline or myo-inositol biosynthetic pathway on cell growth. The recombinant strain BY4742/PRO2 and strain BY4742/INM2 were cultivated in SC-Ura medium in the presence and absence of 0.8 g/L furfural, 3.0 g/L acetic acid and 0.3 g/L phenol. The strain BY4742/pRS426 was performed as the control. Results are the mean of duplicate experiments and error bars indicate SD. [file 13068_2015_329_MOESM7_ESM.pdf]
