# Supplementary material for: Increasing proline and myo-inositol improves tolerance of Saccharomyces cerevisiae to the mixture of multiple lignocellulose-derived inhibitors
Source: Biotechnol Biofuels. 2015 Sep 15;8:142. doi: 10.1186/s13068-015-0329-5 (PMC4570682; doi:10.1186/s13068-015-0329-5)

Figure S7 The growth profiles, glucose consumption and ethanol production of the recombinant strain BY4742/INO1 and its control strain BY4742/pRS426 in SC-Ura medium in the absence of multiple inhibitors. Results are the mean of duplicate experiments and error bars indicate s.d.

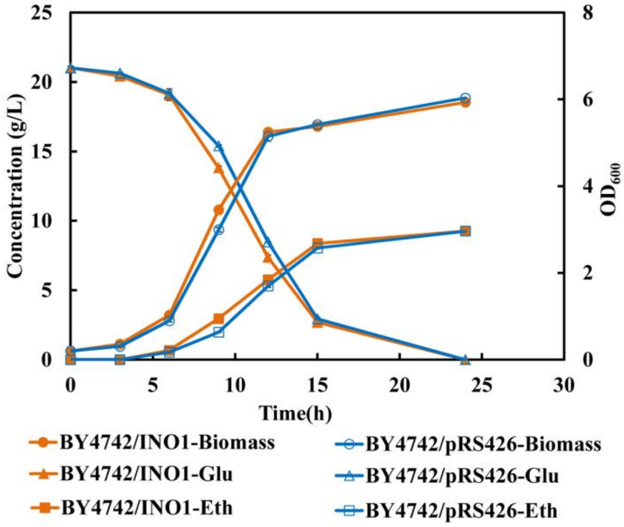

Supplement: Supplementary file 8 — Additional file 8: Figure S7. The growth profiles, glucose consumption and ethanol production of the recombinant strain BY4742/INO1 and its control strain BY4742/pRS426 in SC-Ura medium in the absence of multiple inhibitors. Results are the mean of duplicate experiments and error bars indicate SD. [file 13068_2015_329_MOESM8_ESM.pdf]
