# Supplementary material for: Increasing proline and myo-inositol improves tolerance of Saccharomyces cerevisiae to the mixture of multiple lignocellulose-derived inhibitors
Source: Biotechnol Biofuels. 2015 Sep 15;8:142. doi: 10.1186/s13068-015-0329-5 (PMC4570682; doi:10.1186/s13068-015-0329-5)
Supplement: Supplementary file 9 — Additional file 9: Table S2. Primers used in this study with endonuclease restriction sites underlined and italicized as necessary. [file 13068_2015_329_MOESM9_ESM.pdf]

Table S2 Primers used in this study with endonuclease restriction sites underlined and italicized as necessary

**Primer ID**	**Sequence (5'-3')**
HXT7p_F	CCC*CCCGGG*AGAAGGTTTTGGGACGCTC
HXT7p_R	CG*GAATTC*TTTTTGATTAAAATTAAAAAAACTTTTTG
TEF1t_F	ACGC*GTCGAC*AAATAAGGAGATTGATAAGACTTTTC
TEF1t_R	CC*CTCGAG*GGCTAACTCTCAACAGACAACAAC
PRO1_F	CG*GAATTC*ATGAAGGATGCTAATGAGAGTAAAT
PRO1_R	ACGC*GTCGAC*TCAACGAGGTGGGAATGCC
INO1_F	CG*GAATTC*ATGACAGAAGATAATATTGCTCCA
INO1_R	ACGC*GTCGAC*TTACAACAATCTCTCTTCGAATCTT
INM2_F	CG*GAATTC*ATGGTATTAACGAGGCAAGTACTAGA
INM2_R	ACGC*GTCGAC*TTAGTATTCTAACTCACCCGCAA
PRO2_F1	CG*GAATTC*ATGTCCAGTTCACAACAAATAGC
PRO2_R	cttatcaatctccttatttgtcgacTTATAATGTCACAGTCTTTATATCTAAATCC
PRO2_F2	GGATTTAGATATAAAGACTGTGACATTATAAgtcgacaaataaggagattgataag
